# Efficiency of four trap types and human landing catch in the sampling of *Mansonia* (Diptera, Culicidae) in Porto Velho, Rondônia, Brazil

**DOI:** 10.1371/journal.pone.0315869

**Published:** 2025-01-14

**Authors:** Nercy Virginia Rabelo Furtado, José Ferreira Saraiva, Kaio Nabas Ribeiro, Noel Santos Fernandes Neto, Jéssica Fernanda dos Santos Barroso, Cynara de Melo Rodovalho, Dario Pires Carvalho, Allan Kardec Ribeiro Galardo, José Bento Pereira Lima

**Affiliations:** 1 Laboratório de Entomologia Médica, Instituto de Pesquisas Científicas e Tecnológicas do Estado do Amapá / IEPA, Macapá, Amapá, Brazil; 2 Postgraduate Program in Tropical Medicine, Instituto Oswaldo Cruz, Fiocruz, Rio de Janeiro, Rio de Janeiro, Brazil; 3 Santo Antônio Energia – SAE, Porto Velho, Rondônia, Brazil; 4 Laboratorio de Biologia, Controle e Vigilância de Insetos Vetores (LBCVIV), Instituto Oswaldo Cruz (IOC)/ Fiocruz, Rio de Janeiro, Rio de Janeiro, Brazil; Instituto Leonidas e Maria Deane / Fundacao Oswaldo Cruz, BRAZIL

## Abstract

Entomological surveillance plays a crucial role in designing and implementing mosquito control measures. In this context, developing more effective collection strategies is essential to accurately estimate the entomological parameters necessary for effective control. In this study, we investigated the effectiveness of four traps: CDC light trap, MosqTent, BG-Sentinel, and SkeeterVac, compared to human landing catch (HLC) in the collection of *Mansonia* mosquitoes, known to cause discomfort to riverside populations along the Madeira River in the District of Jaci Paraná, Porto Velho, in Rondônia state, Brazil. Sampling was conducted, during three periods corresponding to two seasons, dry and rainy, over five consecutive days for each period. The captures using HLC and the installation of the traps took place on the grounds of five selected residences from 6 to 10 pm. Rotational exchanges between houses ensured that all traps and the HLC were used in each of the five residences, following a predetermined Latin square pattern. A total of 7,080 mosquitoes were collected, of which 90.5% belonged to the *Mansonia* genus, distributed in four species: *Mansonia titillans* (75.97%), *Mansonia humeralis* (18.91%), *Mansonia amazonensis* (1.90%), and *Mansonia indubitans* (1.37%). HLC captured the highest number of *Mansonia* mosquitoes (58.1%), followed by SkeeterVac (21.8%) and MosqTent (18.9%). CDC and BG-Sentinel showed a very low performance (0.92 and 0.23%, respectively). Although HLC performed better in capturing *Mansonia*, our results suggest that SkeeterVac and MosqTent can serve as valuable additional tools to entomological inventories or sentinels for detecting invasive species in areas with high epidemiological vulnerability, thereby providing evidence-based recommendations for improving mosquito control measures and entomological surveillance.

## Introduction

Culicidae are an extremely important group of insect vectors for public health due to the hematophagous feeding habits of females, which, when feeding, can transmit numerous pathogens that cause human diseases [1, 2]. Among the culicid genera of great medical importance are *Anopheles* Meigen 1818, *Aedes* Meigen 1818, *Culex* Linnaeus 1758, and *Mansonia* Blanchard 1901 [3].

The genus *Mansonia* Blanchard has two subgenera: *Mansonioides* Theobald, with ten species distributed in regions of Asia and Africa, where they are vectors of arboviruses, and *Mansonia*, with 15 neotropical species [4, 5], 12 of which reported for Brazil. These mosquitoes are large and characterised as having adaptations in the respiratory apparatus (siphon and trumpet) in immature stages, enabling them to attach themselves to the roots of aquatic plants and thus obtain oxygen directly from the plant’s aerenchyma [2, 6, 7]. The Mansoniini are specially adapted to develop in lentic environments due to this characteristic in the immature stages, where aquatic macrophytes proliferate, especially the *Pistia stratiotes* Linnaeus 1753 and *Eichhornia crassipes* (Mart.) Solms 1883 [2, 5]. These plants can proliferate in artificial lakes and dams built by companies, creating favourable conditions for the formation of suitable breeding sites for *Mansonia* species [8–10].

*Mansonia* females transmit numerous pathogens that cause human disease [11], such as Eastern [12] and Venezuelan equine encephalitis viruses [13, 14], St. Louis [15] and Japanese encephalitis viruses [16], Rift Valley Fever [17], and filariasis [18]. In Brazil, natural infections have been detected in these mosquitoes, such as Eastern equine encephalitis and Mayaro, and Dengue viruses [12, 19]; however, the vector competence of *Mansonia* species from Brazil has not yet been investigated [19]. Other worrying factors include hematophagous behaviour, which presents strong eclecticism in the choice of blood sources (animals or humans) and can establish bridges between wild zoonotic cycles and rural and urban environments, and aggressive eating habits, which cause great discomfort, making specific locations unsuitable for housing or raising animals for slaughter [2, 5, 20, 21].

Entomological surveillance is a tool for monitoring insect vectors’ biological and ecological characteristics [22]. These data are generated by capturing mosquitoes using different techniques, varying according to the study’s objective and the species of interest [23]. Monitoring *Mansonia* species presents specific particularities due to their biological and behavioural characteristics. The species of this genus present nocturnal and crepuscular behaviour [5], with a preference for attacks outdoors. However, due to this preference for the outdoor environment, they can be found biting throughout the day when people are more active and carry out activities outdoors. In addition, *Mansonia* exhibits intra-domiciliary visiting behaviour when they are at high density [24]. Thus, the capture of adult mosquitoes can be carried out using traps, such as the CDC luminous trap, BG-Sentinel, SkeeterVac, and MosqTent [25], in addition to other models that use light or chemical attractants [26–28].

The human landing catch (HLC) [29] is the most common method used in epidemiological studies [30] and is considered the “gold standard” for capturing hematophagous and anthropophilic insects [25, 30]. Although HLC is widely used, its disadvantage is the need for trained collectors and the supervision of captures, in addition to ethical issues such as collectors’ exposure to mosquito bites that could potentially transmit pathogens [23, 28, 31]. Therefore, the use of traps that do not expose people to the risk of disease contagion and have the same rigour regarding the quality of the samples is of major importance for surveillance studies of insect vectors.

In this study, we compared the effectiveness of four traps commonly used to collect mosquitoes (CDC, MosqTent, SkeeterVac, and BG-Sentinel) in relation to HLC to identify a trap that presents the same effectiveness in collecting *Mansonia* and could replace human attraction, reducing mosquito/catcher contact. Based on the knowledge about *Mansonia* biology and behaviour and the characteristics and mode of action of the traps tested, we hypothesised that MosqTent would perform well and could be a good candidate to replace HLC.

## Material and methods

### Ethics

This project was approved by the Ethical Committee of the Instituto de Pesquisas Científicas e Tecnológicas do Estado do Amapá (CAAE: 88264418.2.0000.0001). The mosquito collections were conducted under the Instituto Chico Mendes de Conservação da Biodiversidade (ICMBio) permit through the Biodiversity Authorization and Information System (SISBIO), number 65279–1.

All professional collectors are authors of the article and have signed a written consent form declaring their qualification to conduct vector capture activities using the HLC and their awareness of the protective measures and risks inherent to the activity, according to the Brazilian guidelines [29]. The residents’ consent was obtained through oral explanation, and everyone accepted that the collections were conducted in their houses.

### Study area

**Fig 1:**
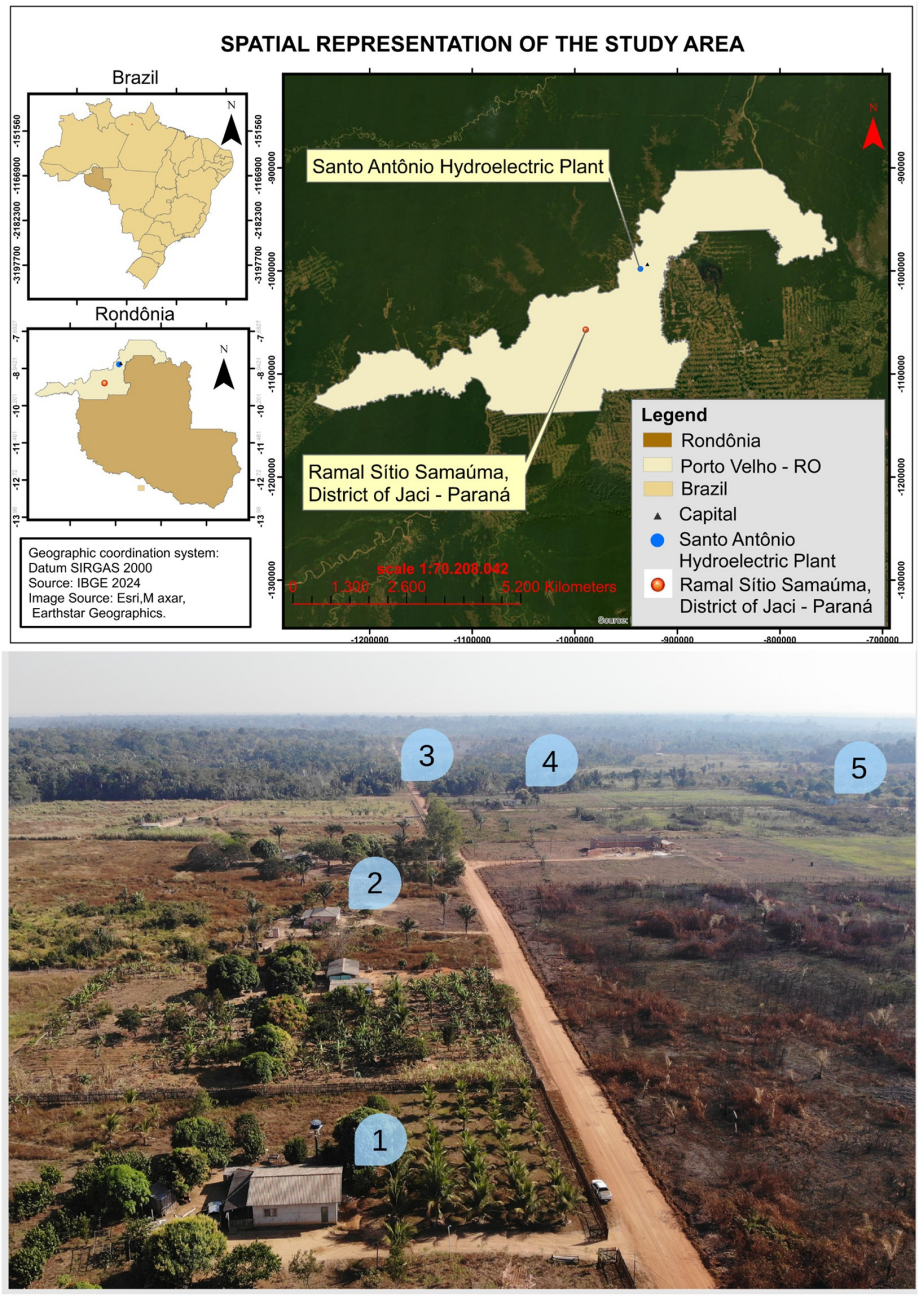
Spatial representation of the study area. Santo Antônio Hydroelectric Power Plant and map of the region, showing the selected locations for evaluating the effectiveness of traps in the District of Jaci Paraná, municipality of Porto Velho, Rondônia, Brazil. Numbers 1–5 correspond to the houses where the tests were carried out. Source: map—IBGE database. Figure—Project image archive.

The study was conducted in the Jaci Paraná District (8.801616°S, 63.950852°W), a rural area of Porto Velho in the state of Rondônia, Brazil. The district is situated on the right bank of the Madeira River, approximately seven kilometers from Porto Velho. Five residences located along Ramal Sítio Samauma, an agricultural region of Rondônia, were selected based on a prior assessment of the presence of *Mansonia* mosquitoes (Furtado et al., unpublished data) (Fig 1). The geographic coordinates of the houses are as follows: House 1 (9.258042°S, 64.441212°W), House 2 (9.256635°S, 64.441462°W), House 3 (9.253039°S, 64.441973°W), House 4 (9.253812°S, 64.441003°W), and House 5 (9.252834°S, 64.438966°W).

According to the Köppen classification, the climate is considered tropical wet (AW), with an average temperature of 21–34 °C and rainfall of 17–264 mm per month. The rainy season runs from October to April, and the dry season from June to August, with transition periods in May and September [32].

### Collection methods

Samplings were carried out in three different periods over five consecutive days. The first and second collection periods were in 2021 (August 2^nd^ to 6^th^ and November 1^st^ to 5^th^) and the third in 2022 (April 11^th^ to 15^th^). The first sampling coincides with the dry season, while the second and third samplings with the rainy season in the region. The average temperature in the area is 25,4 ±1,8 °C, and the relative humidity is 66,4 ±16,9%.

**Fig 2:**
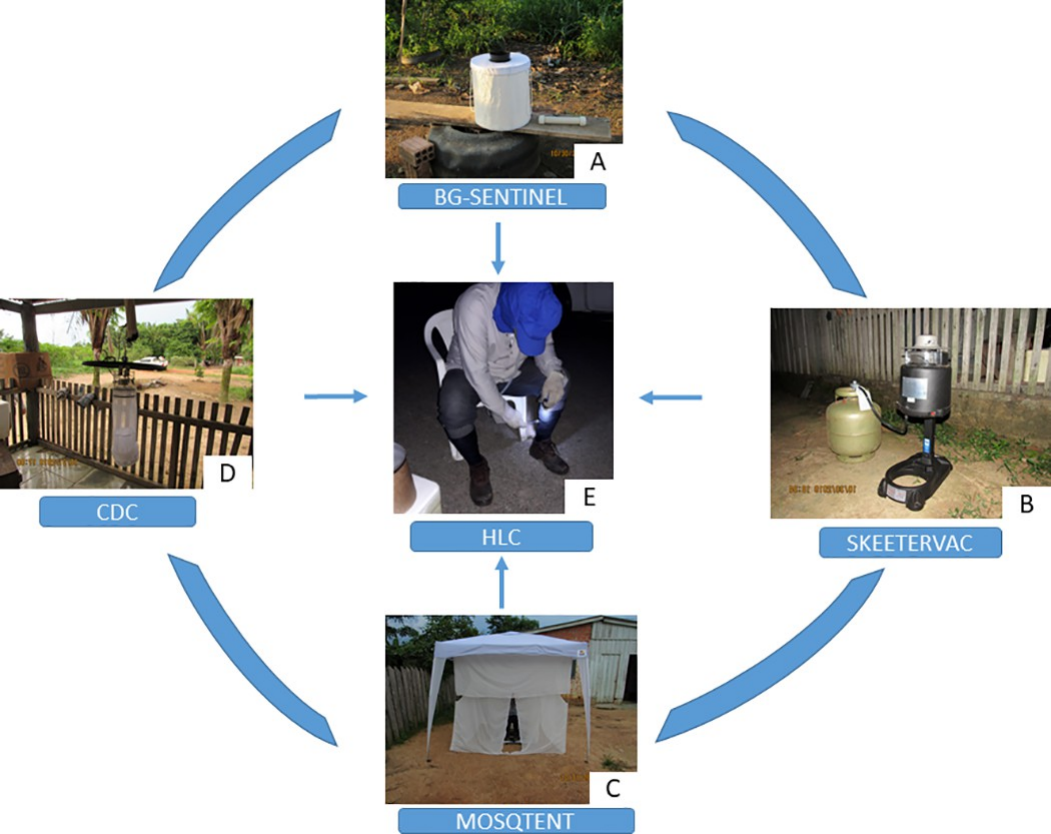
Traps selected for effectiveness evaluation in the area surrounding the Santo Antônio Hydroelectric Power Plant in Porto Velho, Rondônia, Brazil. A: BG-Sentinel; B: SkeeterVac; C: MosqTent; D: CDC; E: HLC. Source: project image archive.

Four traps were selected (CDC model light trap; MosqTent; BG-Sentinel with BG-Lure pheromone; SkeeterVac SV5100, with Lurex and Octenol pheromones as attractants) for comparison with HLC (Fig 2). The choice of traps was based on those commonly used in monitoring culicids. To compare the collection performance of *Mansonia* spp., we standardised the location of trap installation and collection with HLC, always in the outside area of the residences in a dark environment. The MosqTent trap, requiring installation in larger areas, was consistently positioned closest to where HLC and the other traps were installed.

The initial distribution of the methods was random, observing a minimum distance of 132 m to avoid the impact of one trap on the results of the others. When there were no residences at that interval, they were placed at the next house (Fig 1). Each collection event had a rotation, so each methodology was used in all households. The traps were arranged in a Latin square, where each trap was used once in each house per night, from 6 pm to 10 pm, with inspections every hour. For the HLC, the capturer remained seated, with a 5-minute break every hour, collecting during the same period. This collection period was chosen based on the findings of Galardo et al. [24] in a study conducted in the same area, in which the authors observed that the activity peak for *Mansonia* was from 6 pm to 10 pm.

The captured mosquitoes were placed in containers, labelled by trap and time, and kept in a humid chamber, with a 10% sucrose solution offered as a food source until identification. The specimens were killed with ethyl acetate vapours and identified using a stereomicroscope and dichotomous keys by Lane [33] and Barbosa et al. [34]. *Mansonia* mosquitoes with damage to their diagnostic structure were determined to be *Mansonia* spp.

### Data analysis

After taxonomic identification of mosquitoes and compilation of species data, exploratory analyses were conducted on the database, utilising tables and graphs generated with the ggplot2 [35] and dplyr [36] packages. The abundance of mosquitoes captured by each trap was compared to that of the HLC [28]. Additionally, we assessed the species richness captured by the traps relative to the HLC. A nonparametric analysis (Kruskal-Wallis) was performed to verify whether there were significant differences between the abundance of *Mansonia* and other mosquito genera when comparing the different samples and houses. Additionally, a Rate Ratio was calculated to compare the effectiveness of the HLC method with other capture methods, and its associated p-value was assessed to determine the statistical significance of the observed differences.

To evaluate the effectiveness of four traps in capturing *Mansonia* mosquitoes compared to the HLC method, we conducted a generalised linear mixed model (GLMM) analysis. Based on the abundance of mosquitoes collected by each method, the mean and standard errors of the differences in the least squares means associated with the linear mixed model were calculated using Poisson distribution. The abundance recorded by the HLC served as a reference value for comparison, while the house number (in this case, the Latin square number) and the day of collection were treated as independent random variables. The analysis was performed using the lme4 [37] package.

All statistical analyses were performed using R Studio version 3.0.386 (R Studio Team 2023), based on R version 4.2.3 [38]. The significance level for the abovementioned tests was set at p < 0.05.

## Results

During the three sampling periods, 7,080 mosquitoes of 19 species were collected, of which 6,408 were of the genus *Mansonia* (90.5%) and 672 specimens of other Culicidae (9.5%) (Table 1 and S1 Table). In the genus *Mansonia*, the most abundant species was *Mansonia titillans* Walker, 4,868 (68.76%), followed by *Mansonia humeralis* Dyar & Knab, 1,212 (17.12%), *Mansonia amazonensis* Theobald, 122 (1.72%) and *Mansonia indubitans* Dyar & Shannon, 88 (1.24%). A total of 118 (1.67%) of the collected *Mansonia* mosquitoes could not be identified at the species level, as they were damaged (Table 1). The largest number of culicid specimens (38%) was collected in the third sampling, and the first sampling was the one with the highest abundance of *Mansonia*, with 2,485 specimens (Table 1).

**Table 1:** List of species and relative frequency (%) of mosquitoes collected in three sampling periods, using four types of traps and HLC. Sampling was carried out in the outside area of five residences in the District of Jaci Paraná, Porto Velho, Rondônia, Brazil.

Species	Sampling periods						Total	*%*
	1st dry season	*%*	2^nd^ rainy season	*%*	3^rd^ rainy season	*%*		
*Aedeomyia squamipennis*	7	*0*.*28*	1	*0*.*05*	21	*0*.*78*	29	*0*.*41*
*Aedes aegypti*	8	*0*.*32*	0	*0*.*00*	3	*0*.*11*	11	*0*.*16*
*Aedes albopictus*	0	*0*.*00*	1	*0*.*05*	5	*0*.*19*	6	*0*.*08*
*Aedes scapularis*	0	*0*.*00*	68	*3*.*65*	0	*0*.*00*	68	*0*.*96*
*Aedes serratus*	0	*0*.*00*	1	*0*.*05*	5	*0*.*19*	6	*0*.*08*
*Anopheles albitarsis* s.l.	0	*0*.*00*	3	*0*.*16*	0	*0*.*00*	3	*0*.*04*
*Anopheles darlingi*	3	*0*.*12*	0	*0*.*00*	0	*0*.*00*	3	*0*.*04*
*Anopheles mattogrossensis*	2	*0*.*08*	0	*0*.*00*	0	*0*.*00*	2	*0*.*03*
*Anopheles nuneztovari* s.l.	0	*0*.*00*	2	*0*.*11*	0	*0*.*00*	2	*0*.*03*
*Anopheles triannulatus* s.l.	0	*0*.*00*	2	*0*.*11*	0	*0*.*00*	2	*0*.*03*
*Coquillettidia venezuelensis*	4	*0*.*16*	14	*0*.*75*	20	*0*.*74*	38	*0*.*54*
*Culex* sp.	12	*0*.*47*	60	*3*.*22*	308	*11*.*44*	380	*5*.*37*
*Culex* (*Melanoconion*) sp.	0	*0*.*00*	0	*0*.*00*	3	*0*.*11*	3	*0*.*04*
*Culex quinquefasciatus*	6	*0*.*24*	35	*1*.*88*	0	*0*.*00*	41	*0*.*58*
*Limatus durhamii*	0	*0*.*00*	0	*0*.*00*	1	*0*.*04*	1	*0*.*01*
*Mansonia amazonensis*	4	*0*.*16*	12	*0*.*64*	106	*3*.*94*	122	*1*.*72*
*Mansonia humeralis*	674	*26*.*67*	305	*16*.*39*	233	*8*.*66*	1,212	*17*.*12*
*Mansonia indubitans*	48	*1*.*90*	0	*0*.*00*	40	*1*.*49*	88	*1*.*24*
*Mansonia titillans*	1,759	*69*.*61*	1,355	*72*.*81*	1,754	*65*.*16*	4,868	*68*.*76*
*Mansonia* sp.	0	*0*.*00*	1	*0*.*05*	117	*4*.*35*	118	*1*.*67*
*Psorophora albigenu*	0	*0*.*00*	1	*0*.*05*	74	*2*.*75*	75	*1*.*06*
*Psorophora confinnis*	0	*0*.*00*	0	*0*.*00*	1	*0*.*04*	1	*0*.*01*
*Uranotaenia lowii*	0	*0*.*00*	0	*0*.*00*	1	*0*.*04*	1	*0*.*01*
**Total**	**2,527**	***35*.*7***	**1,861**	***26*.*3***	**2,692**	***38*.*0***	**7,080**	***100***

The non-parametric Kruskal-Wallis analysis revealed statistically significant differences in the abundance of *Mansonia* and other Culicidae, with the most pronounced difference observed between the dry and wet seasons (Z = 2.710, p.adj = 0.0201). This significant difference remained when analysing only the abundance of *Mansonia* between the dry and wet seasons (Z = 2.461, p.adj = 0.0415) (S2 Table).

All houses had the same composition of *Mansonia* species, with equal richness of four species. However, there was greater abundance in House 3, with 2,171 (33.88%) specimens, followed by House 1, with 1,838 (28.68%); House 2, with 1,224 (19.1%); House 4, with 788 (12.3%), and House 5, with 387 (6.04%) (S3 Table) (S1 Fig). The number of *Mansonia* mosquitoes collected across the five houses did not show a statistically significant difference (χ^2^ = 4.949, p = 0.2926).

**Fig 3:**
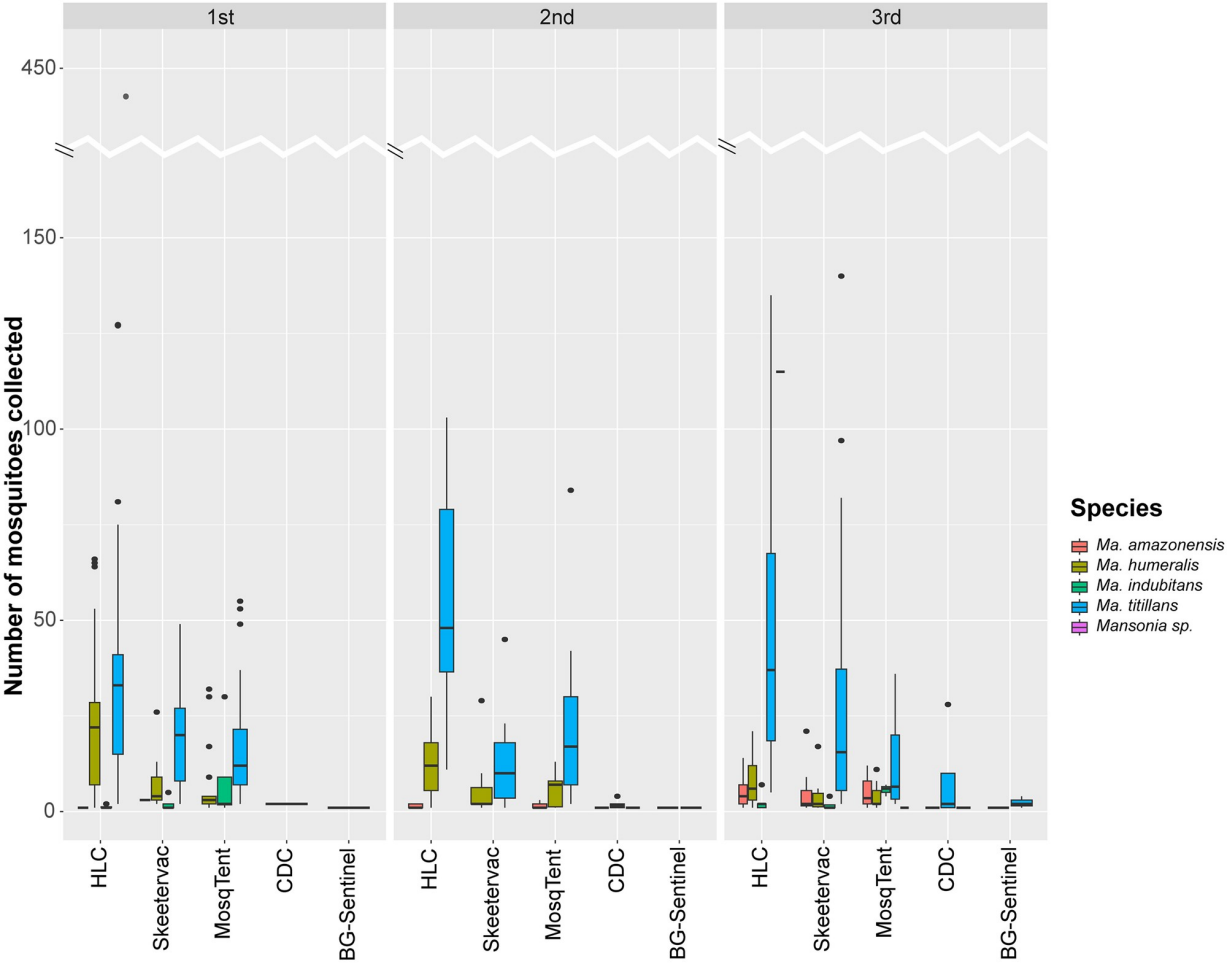
Boxplot of the number of *Mansonia* spp. collected per method in three sampling periods. Samplings were carried out in the surrounding area of Santo Antônio Hydroelectric Power Plant in Porto Velho, Rondônia, Brazil, between 2021 and 2022.

The HLC, SkeeterVac, and MosqTent showed equal richness for *Mansonia*, collecting four species (*Ma*. *amazonensis*, *Ma*. *humeralis*, *Ma*. *indubitans*, and *Ma*. *titillans*), while CDC and BG-Sentinel collected only two species (*Ma*. *titillans* and *Ma*. *humeralis*) (Fig 3 and S1 Fig).

Furthermore, the human capture method (HLC) demonstrated a greater quantity of *Mansonia* spp. collected compared to the four traps tested (Fig 3). In other words, none of the traps came close to the number of mosquitoes collected by HLC (58.1% of the total of *Mansonia*). When comparing performance between traps, SkeeterVac and MosqTent captured the highest quantities of *Mansonia* specimens (21.8 and 18.9%, respectively), while the CDC (0.92%) and BG-Sentinel (0.23%) traps had significantly lower performance (Fig 3). The capture rate for HLC was 21.8, while the capture rate for other methods was 8.54, resulting in a Rate Ratio of 2.55 and a p-value of 0.002, indicating a statistically significant difference.

Table 2 shows the results of the Generalized Linear Mixed Model (GLMM) analysis. The estimated coefficients reflect the average impact of each trap compared to the reference method (HLC). All traps exhibited significant differences regarding the collection efficiency of *Mansonia* spp. when compared to the reference method, as evidenced by statistically significant t-values. According to the performance, the traps can be ordered compared to the reference method, which is determined by the magnitude of the coefficient estimates, with MosqTent and SkeeterVac showing estimates closest to the reference value, followed by CDC and finally BG-Sentinel.

**Table 2:** *Mansonia* collection performance of four traps compared to HLC. The comparison considers the reference value ’Abundance of *Mansonia* collected with HLC’. Linear mixed model fit by REML [‘lmerMod’]. Formula: N ~ method + (1 | day) + (1 | n_home).

Methods	Estimate	s.e.	t value
(Intercept)	28.197	4.047	6.967
BG-Sentinel	-25.204	9.363	-2.692
CDC	-26.774	7.951	-3.367
MosqTent	-17.203	3.877	-4.437
SkeeterVac	-15.225	3.894	-3.910

*N–number of *Mansonia* specimens collected by trap (method), n_home–residence number (1 to 5), and day–days of collection (1 to 15).

**Fig 4:**
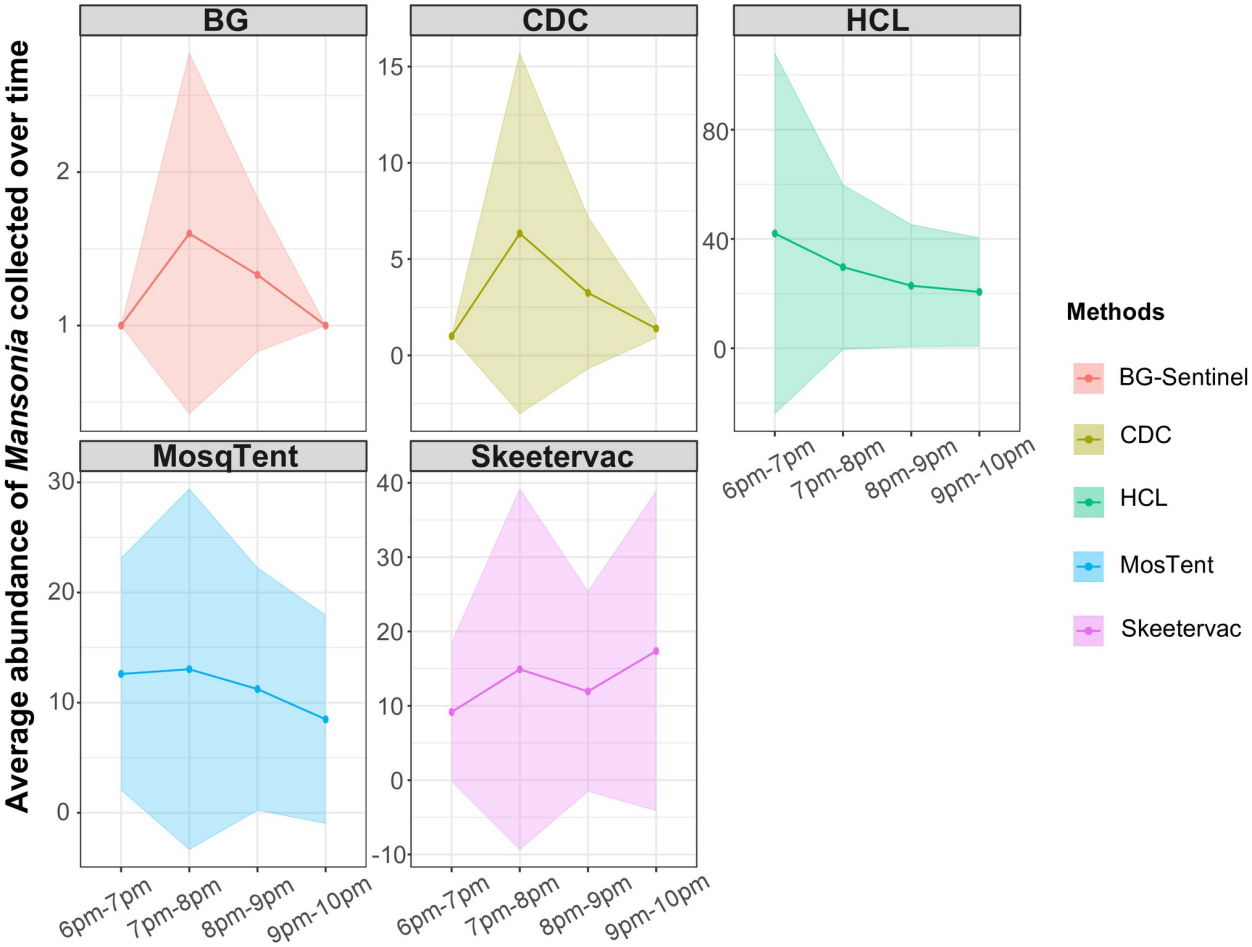
Hourly activity of *Mansonia* spp., by trap and HLC, during sampling periods carried out in the District of Jaci Paraná, Porto Velho, Rondônia, Brazil. The line represents the mean number of mosquitoes sampled at each time point and the shaded area represents the Standard deviation. It is important to note that the y-axis scales vary according to the method.

Regarding the hourly activity of *Mansonia*, HLC recorded peaks in the first hour (6–7 pm). On the other hand, all traps, except for the SkeeterVac, recorded peaks in the second hour (7–8 pm). The SkeeterVac was the only one that did not show a significant drop in mosquito collection in the following hours, maintaining consistent collection over time. Overall, the hourly activity of *Mansonia* in the area was most intense between 6 pm and 7 pm, attributed to the higher number of mosquitoes collected by the HLC, which boosted the number of specimens analysed for the first collection hour (Fig 4).

## Discussion

In the present study, 19 species of mosquitoes were identified, four of which were from the genus *Mansonia*. All collected species have already been recorded in studies in the Brazilian Amazon region [24, 39–44]. The *Mansonia* genus was highlighted in our research due to its aggressive biting behaviour and its high abundance in the studied area, attributed to its proximity to the Madeira River, which concentrates breeding sites for these mosquitoes [45].

Our results show that the abundance peak between *Mansonia* and the other collected culicids does not coincide, with the highest abundance of *Mansonia* being recorded during the dry season (sampling 1). These findings do not corroborate Galardo et al. [24], who identified a greater abundance of *Mansonia* during the transition from the rainy to the dry season. This difference may be related to the collection techniques used. In this study, we used four traps in addition to the HLC that Galardo and colleagues used. A greater abundance of mosquitoes (other than *Mansonia*) was observed during the rainy season, which agrees with previous studies in the region [24, 39–44].

In our collections, *Ma*. *titillans* was the most abundant. Galardo et al. [24] also recorded a high density of this species in Porto Velho. It can be explained by the presence of the preferred macrophyte species (*Pistia stratiotes* and *Eichhornia crassipes*) for laying their eggs in breeding sites close to the collection points [46]. Another critical factor was the time of collection, as *Ma*. *titillans* is a predominantly nocturnal species [24], capable of flying for kilometres in search of food sources and an ideal place for oviposition [47].

*Mansonia* has been characterised as highly eclectic in its choice of blood-feeding hosts and can exhibit intra-household visiting behaviour at high local density [24]. Although our sampling was limited to the surrounding areas of the houses and, therefore, did not allow comparisons of this behaviour, we observed that residences closer to forest areas and with the presence of animals that spend the night in the outside area had a higher density of hematophagous attacks and, consequently, a greater number of mosquitoes collected in the traps and by the HLC technique. This suggests one reason for the higher density of mosquitoes in some houses compared to others [21], although no significant difference was observed.

The number of *Mansonia* captured in the SkeeterVac trap was only smaller than that of the HLC method, proving its efficiency. In comparative studies using Mosquito Magnet (MM), the trap had a better result in collecting culicids when compared to the CDC [48–52]. According to Sant’Ana et al. [52] the MM has a positive relationship with the mosquitoes of the tribes Mansoniini and Sabethini.

In French Guiana, Dusfor et al. [49] observed a higher density of *Anopheles* mosquitoes when using conventional human attraction (HCL) compared to MM and CDC, which was observed for *Mansonia* in the present study. On the other hand, Vezenegho et al. [53] reported that MM traps performed better when associated with pheromones (such as Octenol) in collecting *Anopheles* mosquitoes compared to HLC.

Tent-type traps, such as MosqTent, Human-Baited Double Net Trap (HDN), and Furvela Tent Trap (FTT), were developed to capture mosquitoes using human attractiveness but without endangering the collector, making them an efficient tool to replace HLC [25, 54–56].

Lima et al. [25], when developing and testing the MosqTent, obtained promising results and, despite the HLC’s higher efficiency in the collection of *Anopheles darlingi* Root, 1926, the tent-type trap sampled a greater number of *An*. *marajoara* Galvão and Damasceno, 1942 specimens, which is the secondary vector of *Plasmodium* causing malaria in the state of Amapá.

Tangena et al. [54] observed greater species richness of mosquitoes in HDN traps compared to HLC in Asia. Furthermore, they obtained a similar number of *Anopheles* and *Culex* specimens in the two techniques. Gao et al. [55] showed that HDN is a safe alternative for monitoring *Aedes albopictus* Skuse, 1894.

The BG-Sentinel is an excellent alternative for surveillance of some species of mosquito vectors of pathogens [57], such as those of the *Aedes* and *Culex* genera. It is considered a standard method for surveillance of *Ae*. *albopictus* [58], and when associated with CO_2_, it also collects *An*. *darlingi* in greater quantities than the CDC [27]. However, the results presented here for the collection of *Mansonia* spp. did not demonstrate the same efficiency.

A possible explanation for BG-Sentinel poor performance is that the trap is associated with only one type of pheromone (BG-Lurex). The combination of several odour molecules that mimic human sweat seems more effective in capturing anthropophilic mosquitoes [59], as observed for SkeeterVac, in which Octenol and Lurex were used.

For Alencar et al. [60] and Mello et al. [39], the CDC trap is an excellent tool for collecting Culicidae. In addition, it is efficient in recapturing *Mansonia* species in oviposition areas (macrophyte banks) [40]. However, contrary to what was observed by these authors, the results obtained in the present study do not indicate this trap for collecting *Mansonia*.

Other studies carried out in Porto Velho also found that the CDC trap collected fewer Mansoniini mosquitoes than the Shannon-type trap [39] and fewer *Mansonia* species [61].

Entomological monitoring is necessary to support more effective control strategies [62], especially when the high dispersal capacity of these mosquitoes is considered [47]. Our study investigated the effectiveness of four traps widely used in entomological studies [63] to assess their viability as replacements for HLC, the primary mosquito monitoring technique. One reason is to minimise risks to collectors. Unlike the genus *Anopheles* Meigen, which concentrates its attacks on the lower limbs [64], *Mansonia* manifests diffuse attacks on hosts [65], making its capture challenging even for experienced collectors.

Regarding the *Mansonia* collection performance observed among the tested traps, SkeeterVac obtained a greater quantity of mosquitoes, followed by MosqTent. SkeeterVac’s best performance can be attributed to multiple attractants, such as Lurex and Octenol pheromones, carbon dioxide, heat, and light [66]. Surprisingly, MosqTent, which uses the collector’s attractive signs inside the tent, came second in the number of mosquitoes collected. However, it still significantly outperformed the CDC and BG-Sentinel traps, which use only one attractive signal each, respectively, light and pheromones.

Thus, comparing the effectiveness of the four traps with the HLC revealed that none of them matched the HLC’s capture capacity, both in the quantity and quality of *Mansonia* specimens collected. Despite the risks associated with HLC [67], this technique remains the most reliable for estimating entomological parameters, such as man-hour bite rates and estimates of hourly activity peaks. Furthermore, it is important to highlight the apparent discrepancy observed in the hourly collection rate between the traps and the HLC. Data about activity times can provide valuable additional information for formulating control strategies, especially considering the high eclecticism of *Mansonia* in choosing blood hosts [21], which can keep them active for longer periods in the surroundings of houses, looking for animals that spend the night close to the residences.

Although none of the traps tested in this study achieved similar efficacy to HLC, our results indicate that traps can be complementary surveillance tools. Except for the MosqTent, which requires human presence to attract mosquitoes [25], traps can be installed in remote areas, operating for long periods without interruption [63]. Furthermore, they are useful for obtaining other important entomological parameters, such as species richness and geographic distribution, vector monitoring, and detection of invasive species in places with high epidemiological vulnerability, usually not collected in HLC because the focus of captures is on the target species of the studies. As a perspective, other attractants or the combination of several molecules should be tested to achieve capture results closer to those obtained with human attraction.

## Supporting information

S1 Fig Efficacy of four trap types compared to Human Landing Catch (HLC) in capturing *Mansonia* mosquitoes in the District of Jaci Paraná, Porto Velho, Rondônia, Brazil. The graphic shows the species abundance per house, collected with different traps and HLC.
(TIF)

S1 Table *Mansonia* collection data. Diversity of *Mansonia* adult females captured using different traps and HLC on the grounds of houses in the District of Jaci Parana, Porto Velho, Brazil.
(XLSX)

S2 Table *Mansonia* abundance summary. The table shows statistical analyses between the sampling conducted in two seasons.
(XLSX)

S3 Table Abundance and relative frequency (%) of species of the genus *Mansonia*. The table shows the species of *Mansonia* collected in the outside area of five residences (Houses 1–5) in the District of Jaci Paraná, using four traps and HLC.
(DOCX)
