# Supplementary figures and images for: Efficiency of four trap types and human landing catch in the sampling of Mansonia (Diptera, Culicidae) in Porto Velho, Rondônia, Brazil
Source: PLoS One. 2025 Jan 14;20(1):e0315869. doi: 10.1371/journal.pone.0315869 (PMC11731733; doi:10.1371/journal.pone.0315869)

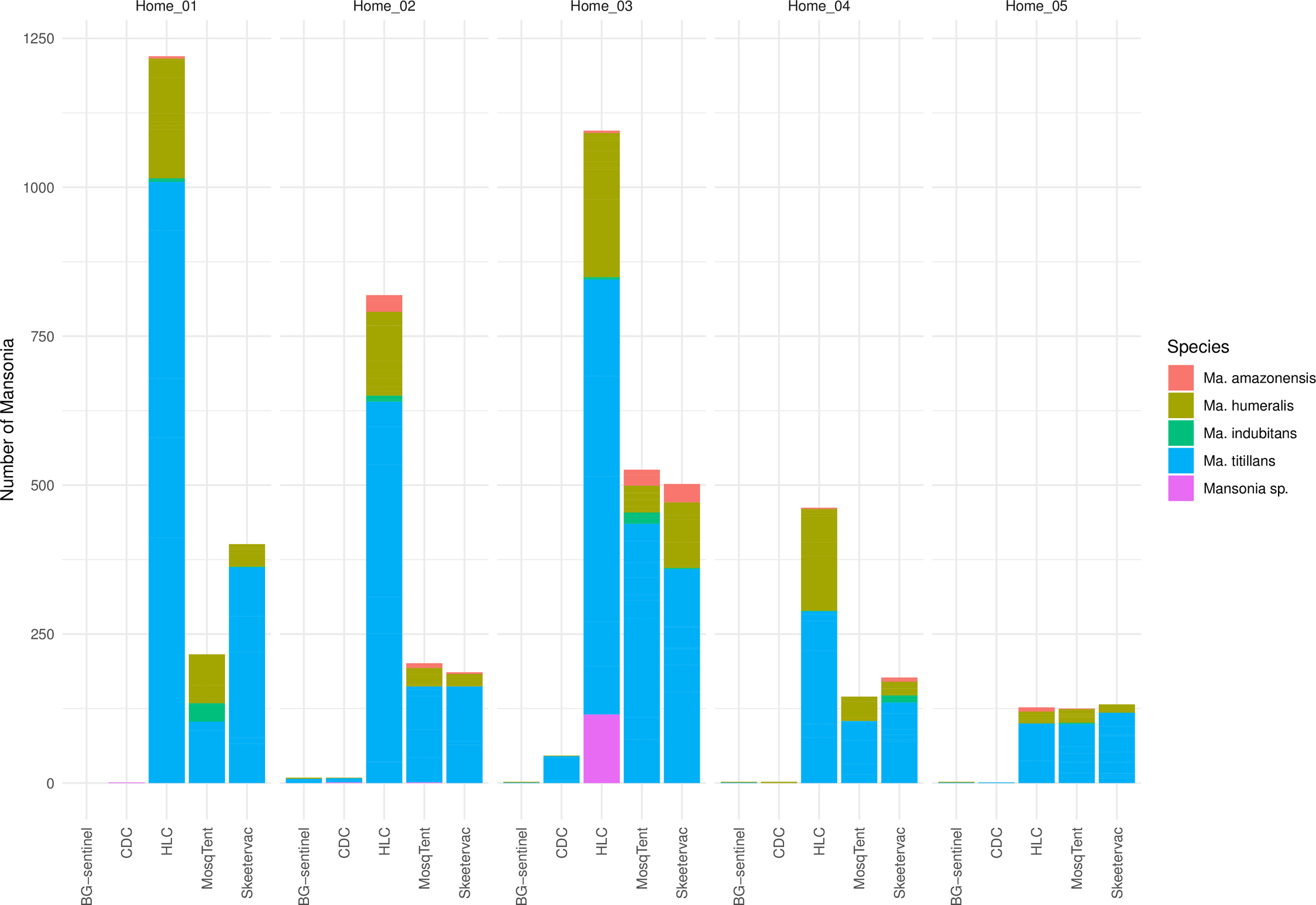

Supplement: S1 Fig — The graphic shows the species abundance per house, collected with different traps and HLC. (TIF) [file pone.0315869.s001.tif]
