# Supplementary material for: Efficiency of four trap types and human landing catch in the sampling of Mansonia (Diptera, Culicidae) in Porto Velho, Rondônia, Brazil
Source: PLoS One. 2025 Jan 14;20(1):e0315869. doi: 10.1371/journal.pone.0315869 (PMC11731733; doi:10.1371/journal.pone.0315869)
Supplement: S3 Table — The table shows the species of Mansonia collected in the outside area of five residences (Houses 1–5) in the District of Jaci Paraná, using four traps and HLC. (DOCX) [file pone.0315869.s004.docx]

**Suple. Table 3. Abundance and relative frequency (%) of species of the genus *Mansonia* collected in the outside area of five residences (Houses 1-5) in the District of Jaci Paraná, using four traps and HLC.**

| **Species** | **House 1** | ***%*** | **House 2** | ***%*** | **House 3** | ***%*** | **House 4** | ***%*** | **House 5** | ***%*** | **Total** | ***%*** |
| --- | --- | --- | --- | --- | --- | --- | --- | --- | --- | --- | --- | --- |
| *Mansonia titillans* | 1,474 | *80.20* | 977 | *79.82* | 1,570 | *72.32* | 528 | *67.01* | 319 | *82.43* | 4,868 | *75.97* |
| *Mansonia humeralis* | 321 | *17.46* | 196 | *16.01* | 399 | *18.38* | 238 | *30.20* | 58 | *14.99* | 1,212 | *18.91* |
| *Mansonia amazonensis* | 4 | *0.22* | 39 | *3.19* | 62 | *2.86* | 9 | *1.14* | 8 | *2.07* | 122 | *1.90* |
| *Mansonia indubitans* | 38 | *2.07* | 10 | *0.82* | 25 | *1.15* | 13 | *1.65* | 2 | *0.52* | 88 | *1.37* |
| *Mansonia* sp. | 1 | *0.05* | 2 | *0.16* | 115 | *5.30* | 0 | *0.00* | 0 | *0.00* | 118 | *1.84* |
| **Total** | **1,838** | ***28.68*** | **1,224** | ***19.10*** | **2,171** | ***33.88*** | **788** | ***12.30*** | **387** | ***6.04*** | **6,408** | ***100*** |
